# Beyond healthy eating: introducing ONI-Hu, the Hungarian version of the Orthorexia Nervosa Inventory

**DOI:** 10.1007/s40519-025-01745-0

**Published:** 2025-04-29

**Authors:** Alexandra Fodor, Balázs András Varga, Adrien Rigó

**Affiliations:** 1 Doctoral School of Psychology, ELTE Eötvös Loránd University, Budapest, Hungary; 2 Institute of Psychology, ELTE Eötvös Loránd University, Budapest, Hungary

**Keywords:** Orthorexia nervosa, Disordered eating, Reliability

## Abstract

**Purpose:**

The aim of this study was to adapt the Orthorexia Nervosa Inventory for use in Hungarian (ONI-Hu), and explore its associations with disordered eating, intuitive eating and mental health measures.

**Methods:**

944 participants completed a test battery, including ONI-Hu, the Three Factor Eating Questionnaire, the Intuitive Eating Scale-2 and the Mental Health Continuum Short-Form. Confirmatory Factor Analysis (CFA) was used to assess the validity of ONI-Hu. Correlation and regression analyses were conducted to evaluate the convergent and discriminant validity.

**Results:**

CFA confirmed the original three-factor structure of ONI-Hu. Positive associations were found between ONI scores and restrictive eating behaviors, and negative associations with intuitive eating measures. Furthermore, the ONI composite factor score displayed no significant relationship with mental health indicators.

**Conclusions:**

ONI-Hu exhibits strong reliability and validity, and provides a deeper understanding of ON. Results suggest that orthorexic behaviors may serve as a coping mechanism, offering an illusion of control and emotional security. Inconsistent findings about the relationship between ON tendencies and mental health indicators propose that the sense of control might provide a false sense of well-being to the individual, distorting their perceptions of their overall health.

*Level of evidence* Level V, descriptive cross-sectional study.

## Introduction

Orthorexia nervosa (ON) often begins as an attempt at a lifestyle change to improve health, driven by factors like chronic illness diagnosis [1], or eating disorder recovery [2]. Later, the individual's thoughts, emotions, and behavior center around healthy eating. One with ON labels food as “healthy, pure, right” or “unhealthy, impure, wrong” [3] and avoids specific ingredients, such as gluten or sugar [4].

Despite an increasing number of people affected with high ON tendency, ON is not yet classified as a mental disorder and, therefore, is not included in the DSM-5 or the ICD either [1]. This means it cannot be officially diagnosed even if someone exhibits a high tendency to ON. Therefore, when we refer to someone as having orthorexia, it does not indicate a formal diagnosis established by a qualified professional but rather a tendency towards the characteristics of orthorexia.

A recent proposal about possible ON diagnostic criteria includes beliefs and rigid rules related to healthy eating and adherence to a nutritionally unbalanced diet [5]. It also describes the emotional, cognitive, and psychosocial impairments related to the disorder (such as anxiety, guilt, problems with concentration, or low self-esteem). Individuals with ON frequently experience emotional distress. Consuming food considered unhealthy is typically followed by feelings of guilt, chronic worry, fear of deteriorating health, and even self-punishing behavior, where they further restrict their diet and even engage in strict fasting[3, 6]. The proposed diagnostic criteria also include problems with physical health (malnutrition, low body weight, gastrointestinal and hormonal symptoms) and psychosocial impairments [5].

Individuals with orthorexic tendencies often experience anxiety, distress, or dissatisfaction with their lives. Controlling what they eat and pursuing rigid dietary rules may give the illusion of control and provide a sense of identity or competency [7, 8]. The rigid dietary rules and obsession with food purity may also lead to a decreased capacity or desire to engage in social activities [9]. Research also suggests that individuals with orthorexic tendencies experience stronger anxiety in social situations that involve food, which could, again, cause the avoidance of social events centered around meals [10]. Social isolation often negatively affects interpersonal relationships and may compromise overall quality of life [11].

Orthorexia has been consistently associated with disordered eating behaviors and attitudes [12]. Fundamentally, a history of dieting may contribute to the development of disordered eating patterns and obsession with health [13, 14]. Furthermore, restricting high-calorie foods has been linked to orthorexic tendencies [15]. Additionally, a higher prevalence of disordered eating attitudes has been associated with more significant orthorexic tendencies [16–19]. Findings also indicate that cognitive restraint of eating, uncontrolled eating, and emotional eating behaviors are positively associated with orthorexic tendencies. In contrast, uncontrolled eating and emotional eating are negatively related to healthy eating patterns [20]. Also, individuals reporting orthorexic symptoms share more similarities with those with elevated levels of disordered eating compared to those with fewer eating disturbances in the general population [21].

Intuitive eating (IE) refers to having a strong connection with hunger and satiety cues and determining when and how much to eat in response to these internal signs [22]. Those with a high tendency to intuitive eating tend to choose foods they enjoy while considering what helps their bodies function well [22]. IE has been consistently found to be associated with lower levels of eating disorder symptoms, restrictive eating, body image concerns, and psychological distress and is positively related to psychological well-being, self-esteem, social support, and quality of life [22–29].

Also, those who showed increases in intuitive eating during psychological treatment, improved more in their eating disorder symptoms [30]. Research suggests that intuitive eating may be protective against orthorexic tendencies. Individuals who tend to focus more on their body's signals and functions are more likely to show more appreciation toward their body. Those who appreciate their body tend to be more attuned to its needs and internal signals and more likely to honor them rather than be governed by external factors, such as rules about eating [31].

ON has overlapping characteristics with two psychiatric disorders: anorexia nervosa (AN) and obsessive–compulsive disorder (OCD) [3]. Despite of their similarities, a significant difference between AN and ON is that individuals with AN focus on the quantity of the food, while ON centers around its quality [3, 4]. The similarity between ON and OCD manifests in the intrusive thoughts and ritualized behaviors—however, ON is egosyntonic, and OCD is an egodystonic disorder [3].

The lack of proper diagnostic criteria or reliable screening tools and the socially desirable nature of healthy eating make it challenging to determine the prevalence of ON. The most widely used scale is the ORTO-15 [32] and its adapted versions (in Hungary, the ORTO-11-HU [33]). The tool helped the researchers a lot in investigating ON, and the translated versions made it possible to examine the disorder across different countries and cultures, analyze its correlates, and get more detailed information on ON.

However, concerns about the reliability and validity of ORTO-15 and its derivatives have been raised in various studies. The findings on ORTO-15 suggest that the questionnaire has difficulties in differentiating healthy and pathologically healthy eating, possibly leading to identifying dieting as harmful [34–36]. Also, there are items in ORTO-15 that are similar to some items in the EAT-26 questionnaire, measuring dieting. Also, the recently proposed diagnostic criteria include physical impairments related to ON; the ORTO-15 does not cover this aspect [5, 35].

Furthermore, studies using the ORTO-15 have reported highly variable prevalence rates of orthorexia, ranging from 6.9% to over 80% [32]. Furthermore, a study using ORTO-15 found that 80% of participants did not restrict their food choices, yet over 70% scored as having ON, with fever than 1% experiencing medical complications [34]. These results suggest that the ORTO-15 may overestimate the prevalence of orthorexia. Also, according to a review, studies using ORTO-15 reported inconsistent Cronbach Alpha scores, with a mean Alpha of 0.55, falling below the recommended standards for research (0.7) and diagnostic use (0.9) [37].

In addition, adapted versions of ORTO-15 and its derivatives, including 11- and 9-item versions, exhibited diverse factor structures [38], and had psychometric limitations, such as lacking a good model fit or having low internal reliability scores [39, 40]. The findings suggest that ORTO-15 may not be optimal for assessing or diagnosing ON. Alternative measurement tools, such as the Dusseldorf Orthorexia Scale [41] and the Eating Habits Questionnaire [42] demonstrate higher reliability and include items related to preoccupation, compulsive behavior, emotional distress and psychosocial impairments. However, they all lack items assessing physical impairments related to orthorexic tendencies.

The Orthorexia Nervosa Inventory (ONI) was developed on purpose to create a measurement tool assessing the 4 main aspects of ON [43]. The authors aimed to establish a four-factor structure, however, the items measuring psychosocial and physical impairments ended up loading on a single factor, which resulted in the development of the three-factor model, which includes Psychosocial and Physical Impairments (Impairments), Behaviors and Preoccupation (Behaviors) and Emotional distress (Emotions) [43].

The overall internal consistency of the original tool was 0.94, and for the three ONI subscales, Cronbach alpha varied from 0.88 to 0.90 [43]. ONI has already been adapted to Italian and Turkish. Both studies confirmed the original three-factor structure and demonstrated strong internal consistency and validity (reliability indicators varied between 0.91 and 0.94 for the overall scale and between 0.81 and 0.81 for the three subscales) [44, 45].

ONI was associated with healthy eating behaviors; each scale correlated positively with vegetable and fruit consumption and correlated negatively with meat, dairy, and sweets consumption [43]. ONI scores were higher if the individual had a diagnosis of anorexia, bulimia, or binge eating disorder versus if they did not have any of these. Overall ONI scores and each of the scales were positively associated with obsessive tendencies, and they were also higher among those who had a prior OCD diagnosis [43].

Interest in ON emerges in Hungary as well. However, there are still gaps in the literature, and most of the studies used the ORTO 11-HU questionnaire (a derivative of ORTO-15). The present study aims to describe the adaptation process of Orthorexia Nervosa Inventory—Hungarian Version (ONI-Hu), its psychometric properties, and the factor structure of the original ONI. We analyzed the relationship between ON characteristics, disordered eating behavior, and mental health measurements. We hypothesized ON scores to have a positive association with restrictive eating, psychological and emotional well-being, and a negative association with uncontrolled eating, emotional eating, intuitive eating, and social well-being.

## Method

### Participants

84.4% of the 944 participants were females, and the others were males. The mean age of the participants was 31.8 years. Half of the participants were college or university graduates, and over 30% completed high school education. Based on self-reported measures, the average BMI of the respondents was 24.59. Most of them fell into a healthy weight range (18.5 ≤ BMI ≤ 24.9), and 19.3% were overweight (25 ≤ BMI ≤ 29.9). 7.9% were underweight (18.49 ≥ BMI), and 10.5% were obese (BMI ≥ 30). 13.1% of the respondents followed gluten-free, 14.9% followed the lactose-free diet, 8.2% were vegetarian, and 7.5% were vegan (see Table 1).

**Table 1:** Basic demographics of participants

Variables	n(%) or mean ± SD
Age (years)	31.8 ± 12.5
BMI	24.59 ± 14
Gender	
emale	797 (84.427)
Male	147 (15.572)
Marital status	
Single	276 (29.2)
In a relationship	394 (41.7)
Married	244 (25.8)
Divorced	20 (2.1)
Widowed	10 (1.1)
Level of education	
Primary school	23 (2.4)
Vocational school	18 (1.9)
High school	322 (34.1)
Non-university degree	59 (6.3)
University	477 (50.5)
Postgraduate/doctored	45 (4.8)
Special diet	
Gluten-free	124 (13.1)
Lactose-free	141 (14.9)
Dairy-free	105 (11.1)
Soy-free	53 (5.6)
Vegetarian	77 (8.2)
Vegan	71 (7.5)
No_diet	447 (47.4)

## Instruments

### Orthorexia nervosa inventory

The Orthorexia Nervosa Inventory [43], containing 24 items, requires participant responses on a 4-point Likert scale (1 = not at all true; 4 = very true). The original version includes three factors: assessing ON-related Behaviors, Emotions, and Impairments. The ONI-Hu adaptation process involved multiple steps: an initial translation into Hungarian by an independent psychology student and a back-translation into English by another student. The authors reviewed the re-translated version, resulting in a few modifications. Subsequently, a psychologist conducted the final re-translation into Hungarian (Table 2). For the internal consistency of Cronbach alpha and McDonald’s omega scores, see Table 3.

**Table 2:** Factor_loadings

	Estimate	S.E	Est/S.E	Two-Tailed *P*-Value
**Impairments**				
10. Health professionals have expressed concern that my diet is too restrictive. (Jelezte már nekem egészségügyi szakember, hogy aggódik értem az étrendem szigorúsága miatt.)	0.800	0.063	11.524	0.000
24. The stricter I become with my diet, the more I seem to experience one or more physical symptoms, such as fatigue, faintness, heart racing, nausea, diarrhea, pain, etc. (Minél szigorúbban diétázom, annál többször érzem az alábbi fizikai tünetek valamelyikét, vagy többet is: fáradtság, szédülés, szapora szívverés, hányinger, hasmenés, fájdalom.)	0.641	0.044	16.683	0.000
12. My cleanses or fasts have become more frequent or severe over time. (Egyre gyakrabban és gyakrabban koplalok vagy tartok tisztítókúrát.)	0.698	0.052	13.295	0.000
5. My food restrictions have led me to lose more weight than people would say is good for me. (Csak bizonyos ételeket eszem meg, ami miatt csökkent a súlyom – és ez a családom szerint már nem egészséges.)	0.825	0.040	20.775	0.000
19. Whenever I feel sick, family or friends comment, that the illness may be because my diet is too restrictive. (Ha megbetegszem, a családom és a barátaim meg vannak róla győződve, hogy a szigorú diétám miatt történt.)	0.744	0.046	16.286	0.000
14. Even though I have eaten much healthier over time, my physical health has actually declined. (Annak ellenére, hogy egyre egészségesebben eszem, a testi egészségem romlik.)	0.719	0.041	17.690	0.000
3. As a result of the amount of time I devote to my healthy diet, I have spent less time than I used to with my family or friends. (Annyira sok időt vesz el tőlem az egészséges étkezés, hogy emiatt kevesebb időt töltök a barátaimmal és a családommal, mint korábban.)	0.767	0.042	18.322	0.000
20. While spending time with family or friends, I am frequently distracted by thoughts of eating healthily. (Ha a barátaimmal vagy a családdommal vagyok, gyakran elterelik a figyelmem az egészséges étkezéssel kapcsolatos gondolatok)	0.732	0.042	17.414	0.000
7. My healthy eating is a significant source of stress in my relationships. (Az egészséges étkezésem gyakran okoz feszültséget a kapcsolataimban.)	0.773	0.034	22.479	0.000
16. As a result of the amount of time I devote to my healthy diet, I have either missed time at work or missed classes at school. (Annak eredményeképp, hogy ennyi időt foglalkozok az egészséges étkezéssel, előfordult már, hogy nem mentem dolgozni, vagy kihagytam tanórákat az iskolában.)	0.766	0.063	12.111	0.000
Behaviors				
17. I either do not buy processed food products or I compulsively check the nutrition labels to ensure that only healthy and pure ingredients are included. (Egyáltalán nem vásárolok feldolgozott ételt, vagy ha igen, akkor aprólékosan ellenőrzöm, hogy csak egészséges és tiszta hozzávalókat tartalmazzanak.)	0.665	0.031	21.358	0.000
11. I follow a healthy diet with many rules.( Egészséges étkezést követek, ami kapcsán számos szabályt állítottam fel magamnak.)	0.788	0.022	35.549	0.000
8. Over time, my diet has come to include elimination of entire food groups that I believe are unhealthy. (Az étkezési szokásaim fokozatosan azt eredményezték, hogy ma már egész ételcsoportokat hagyok ki az étkezéseimből, mert egészségtelennek gondolom őket.)	0.773	0.027	28.828	0.000
6. Preparing food in the most healthful way is very important in my diet. (Nagyon fontos része az étkezési szokásaimnak, hogy az ételeket a lehető legegészségesebb módon készítsem el.)	0.733	0.026	28.576	0.000
22. I strictly avoid all foods I feel are unhealthy. (Mindennemű olyan ételt szigorúan elkerülök, amit nem tartok egészségesnek.)	0.847	0.024	36.023	0.000
18. The number of healthy dietary rules that I follow has progressively increased over time. (Az egészséges étkezésre vonatkozó elveim, szabályaim száma az idő elteltével nagy mértékben megnőtt.)	0.731	0.028	25.844	0.000
15. Healthy eating is among the most important things in my life. (Az egészséges étkezés életem egyik legfontosabb eleme.)	0.839	0.021	40.532	0.000
4. I follow a health-food diet rigidly, only eating what my diet allows and not allowing myself any deviations from this diet. (Szigorúan csak olyan ételeket eszem, amiket egészségesnek tartok, és ettől egyáltalán nem szoktam eltérni.)	0.814	0.024	33.572	0.000
2. I care much more about the healthiness of what I eat than the pleasurable taste of food. (Számomra fontosabb, hogy az étel, amit eszek egészséges legyen, mint az hogy finom.)	0.630	0.032	19.403	0.000
Emotions				
9. When I stray from my healthy diet, I can only think about what a failure I am. (Ha nem eszem egészségesen, kész csődnek érzem magam.)	0.898	0.018	51.678	0.000
13. Whenever I eat anything unhealthy, I feel a great sense of personal impurity. (Ha egészségtelen ételt eszem, nagyon szégyellem magam.)	0.754	0.019	43.926	0.000
1. I feel much guilt or self-loathing when I stray from my healthy diet. (Nagy mértékű bűntudatot és önutálatot érzek, ha nem eszem egészségesen.)	0.793	0.022	35.104	0.000
23. Feeling good about my body is completely dependent on me strictly following my healthy diet. (Az, hogy hogyan érzem magam a testemben az teljes mértékben attól függ, hogy mennyire eszem egészségesen.)	0.842	0.031	24.603	0.000
21. Just the thought of me eating something unhealthy makes me very anxious. (Már a gondolata is szorongással tölt el annak, hogy valamit egészségtelent egyek.)	0.912	0.025	35.250	0.000

**Table 3:** ONI relationships with eating behaviors and mental health (Spearman’s correlation)

Variables	(1)	(2)	(3)	(4)	Cronbach Alpha	McDonald’s Omega	Skewness	Kurtosis
(1) ONI Behaviors	–				0.88	0.89	0.79	− 0.08
(2) ONI Impairments	0.49***	–			0.8	0.81	2.76	9.03
(3) ONI Emotions	0.51***	0.48***	–		0.83	0.85	1.41	1.72
(4) ONI composite factor	0.92***	0.68***	0.75***	–	0.91	0.91	1.17	1.32
(5) TFEQ Uncontrolled eating	− 0.11***	0.16***	0.26***	0.06	0.86	0.86	0.55	0.02
(6) TFEQ Cognitive restriction	0.44***	0.36***	0.43***	0.50***	0.8	0.81	0.13	− 0.64
(7) TFEQ Emotional eating	− 0.08*	0.10**	0.24***	0.06	0.94	0.94	0.76	0.23
(8) Intuitive Eating Scale (SUM)	− 0.03	− 0.20***	− 0.37***	− 0.19***	0.86	0.87	− 0.39	0.14
(9) IES Unconditional Permission to Eat	− 0.58***	− 0.40***	− 0.55***	− 0.64***	0.76	0.78	− 0.28	− 0.35
(10) IES Eating for Physical Rather than Emotional Reasons	0.15***	− 0.05	− 0.20***	0.01	0.9	0.91	− 0.36	− 0.55
(11) IES Reliance on Hunger and Satiety Cues	0.02	− 0.12***	− 0.16***	− 0.08*	0.84	0.84	− 0.49	0.7
(12) IES Body-Food Choice Congruence	0.54***	0.11***	0.11**	0.40***	0.85	0.86	− 0.49	0.51
(13) MHC Social well-being	0.07*	− 0.08*	− 0.17***	− 0.03	0.75	0.76	0.15	− 0.59
(14) MHC Emotional well-being	0.11***	− 0.11***	− 0.17***	− 0.02	0.88	0.88	− 0.56	− 0.46
(15) MHC Psychological well-being	0.13***	− 0.11**	− 0.20***	− 0.01	0.88	0.88	− 0.58	− 0.33

*** p < 0.01, ** p < 0.05, * p < *0.1 with Bonferroni correction*

## Eating behaviors

### Three factor eating questionnaire

The Three Factor Eating Questionnaire [46] was translated and validated into Hungarian in 2010 [47]. The questionnaire has 21 items, measures disordered eating, and includes three factors: Uncontrolled Eating, Emotional Eating, and Cognitive Restraint. Participants must indicate how accurate the statements are on a 4-point Likert scale for 20 items (1 = definitely true; 4 = definitely false for items 1–16 and 1 = definitely false;4 = definitely true for items 17–20), and on an 8-Point Likert scale (1: I eat what I want, whenever I want, 8: I restrict my eating) for the last item. Items 1–16 and 21 are reverse coded; the total score ranges from 22 to 92. For the internal consistency Cronbach alpha and McDonald’s omega scores, see Table 3.

### Intuitive eating scale-2

The Intuitive Eating Scale-2 (IES-2) was also used to measure eating behaviors. IES-2 evaluates the individual’s tendency to rely on their body’s cues (e.g., hunger and satiety) when eating, thus measuring an adaptive eating behavior [48]. The IES-2 was developed to overcome the limitations of the original IES [48]. The questionnaire was translated and adapted to Hungarian in 2021 [49]. IES-2 contains 23 items, and the participants must indicate how accurate the statements are on a 5-point Likert scale (1 = strongly disagree, 5 = strongly agree). Items 1–3 and 7–10 are reverse coded. The IES2 includes four subscales: Unconditional Permission to Eat (UPE), Eating for Physical rather than Emotional Reasons (EPR), Reliance on Hunger and Satiety Cues (RHSC), and Body-Food Choice Congruence (BFCC) (Tylka, Kroon Van Diest, 2013). For the internal consistency Cronbach alpha and McDonald’s omega scores, see Table 3.

### Mental health and well-being

We measured mental health indicators with the Mental Health Continuum-Short Form [50], which was translated and validated to Hungarian in 2020 [51]. The questionnaire has 14 items and three subscales: Emotional, Social, and Psychological Well-Being. Participants must respond to statements on a 6-point Likert scale (0 = Never, 5 = Every Day) with no reversed items. One way to evaluate the answers is to determine the total score (ranging from 0 to 70) and the scores of the three subscales. A higher score indicates a higher level of well-being. For the internal consistency Cronbach alpha and McDonald’s omega scores, see Table 3.

### Procedure

The study was approved by the Ethics Committee of Eötvös Loránd University (Registration number: 2020/461). We recruited participants from various sources to have a large sample size—from universities in Hungary and from social media (such as reaching out to online groups related to healthy eating). We collected our data online using an online test battery on Qualtrics. The respondents completed an informed consent form, a demographic questionnaire, and a question about following a specific diet or not and proceeded to fill out the Hungarian version of the Intuitive Eating Scale-2 (IES-2) [48], the Three Factor Eating Questionnaire (TFEQ) [46], Orthorexia Nervosa Inventory (ONI-Hu), and the Mental Health Continuum Short-Form (MHC) [50]. The questionnaires and the questionnaire items were presented in a fixed order.

### Planned analyses

This study aimed to confirm the factorial structure of the Orthorexia Nervosa Inventory on a Hungarian Sample. We analyzed data in Stata 14.0 [52] and MPLUS [53]. To confirm the factorial structure of the Orthorexia Nervosa Inventory on a Hungarian Sample, we used Confirmatory Factor Analysis (CFA). Since the data were categorical, CFA was performed with Weighted Least Squares with Mean Variance and Adjusted Statistics Estimator (WLSMV). Our sample size met the recommendation since it exceeded 200 [54]. We relied on the following rule of thumb for sample size estimation: we aimed to ensure 5–10 observations per estimated model parameter. Based on this approach, the minimum required sample size for the applied model was 270 participants, with a recommended size of 540. Since we could not determine the factor loadings before conducting the CFA, we aimed to approach this recommended sample size. Participation was mandatory; therefore, for respondents who dropped out, we can refer to this as attrition. We examined the randomness of this attrition using a statistical test. Little's Missing Completely at Random (MCAR) test was nonsignificant, indicating that the missing data were random. Consequently, we applied the listwise deletion procedure.

Model fit was considered acceptable if CFI and TLI values were more significant than or close to 0.95, or > 0.90, and RMSEA ≤ 0.05 with the 90% confidence interval < 0.08 [55]. Factor loadings of at least 0.6 were considered appropriate.

Additionally, modification indices were examined to identify potential relationships between variables that could improve the model fit. The study also aimed to analyze other disordered eating patterns and levels of mental health and well-being related to orthorexic tendencies. Correlation and regression analyses were performed to assess convergent and discriminant validity. Since the normality of the ONI factors and the pooled factor were not met (all Shapiro–Wilk tests were significant), a robust correlation procedure (Spearman) was used for correlation analysis, with p-values adjusted using Bonferroni correction to control for family-wise error. In this case, the regression model is part of the validity test, where the explanatory variables are included as covariates in the model. It plays less of a causal role and more of a relational role. Thus, the model offers a more complex analysis, as the covariates included control for each other's effects, making their individual effects more straightforward than if we only performed a correlation analysis. The model provides information on the impact of some demographic variables (sex and age) and the variables included in the correlation analysis.

## Results

### Factorial structure of the ONI

CFA results indicated that the fit of the three-factor structure is adequate since both absolute and relative fit indices fell within the expected ranges (χ2 (249) = 929.662*, RMSEA = 0.067, CI90% = 0.062–0.072, CFI = 0.932, TLI = 0.924). The factor loadings were high, all above 0.60 overall, with standardized beta values ranging from 0.75 to 0.91 for Emotions, 0.64 to 0.86 for Behaviors, and 0.66 to 0.84 for Impairments (see Table 2). After the CFA, the modification indices were examined, which showed no covariance that would significantly improve the model's fit. The inclusion of a correlation between individual items is not justified.

We observed significant correlations among the individual factors in all cases, the highest one between Emotions and Impairments at 0.71, followed by Behaviors and Impairments at 0.71, and the lowest between Emotions and Behaviors at 0.57, all the considered large effect sizes. However, due to the low number of male respondents and the absence of responses in specific scale categories within the male group, invariance analysis could not be performed on the CFA models. Given these limitations, we incorporated demographic variables into the models instead of conducting an invariance analysis to account for potential differences. We examined the impact of demographic variables on the ONI by using a regression model.

Regarding the internal reliability indicators, both the reliability of the ONI Composite and the subscales proved to be good, similar to the other questionnaires used in the analyses (Cronbach’s alpha and McDonald’s omega values can be found in Table 3).

### Convergent and discriminant validity

The weakest (medium) correlation was between ONI_Behaviors and ONI_Impairments (ρ(942) = 0.485, p < 0.001, [CI95%: 0.434–0.532]), while the highest correlation was between ONI_Behaviors and ONI_Emotions (ρ(942) = 0.512, *p* < 0.001, [CI95%: 0.463–0.557]). The strongest correlation of the composite scale is with ONI_Behaviors (ρ(942) = 0.916, *p* < 0.001, [CI95%: 0.915–0.926]), suggesting a possible conceptual identity *(*Table 3*)*.

The ONI composite factor scale exhibits appropriate covariance with the variables used to test validity, and several negligible correlations confirm the discriminant validity of ONI-Hu. Examples of small effect sizes or nonsignificant relationships include Uncontrolled Eating, Emotional Eating, Eating for Physical Rather than Emotional Reasons, and all three MHC subscales (see statistical data in Table 3). Furthermore, the ONI composite factor showed no statistical relationship with the MHC total factor. Regarding the MHC subscales, the analysis showed both positive and negative associations.

ONI composite factor scale demonstrated a moderately strong, positive correlation with the Cognitive Restriction subscale and a strong negative correlation with the Unconditional Permission to Eat subscale, supporting convergent validity.

### Regression analysis of ONI composite factor with the variables included in the validation process

A regression analysis was conducted to assess the impact of covariates. Due to multicollinearity, IES-2 total score was excluded from the analysis as the VIF indicator exceeded 3. The regression model was significant (F(17,904) = 51.26, *p* < 0.001), with an overall adjusted R^2^ of 49.1% (Table 4).

**Table 4:** Regression analysis

Orthorexia_Nervosa_Inventory (ONI)—composite factor		Coef	St.Err	Std. Beta	t-value	p-value	[95% Conf	Interval]	Sig
Sex		0.037	0.029	1.296	0.195	0.032	− 0.016	0.080	
Age		0.003	0.001	2.862	0.004	0.075	0.024	0.126	***
Base: 8 overall or less									
Vocational_school/technical_school		0.011	0.100	0.109	0.913	0.025	− 0.432	0.483	
High_school		− 0.039	0.067	− 0.577	0.564	− 0.091	− 0.399	0.217	
Higher_education at non-university level		− 0.042	0.078	− 0.535	0.593	− 0.098	− 0.456	0.260	
College/university degree		− 0.090	0.067	− 1.347	0.178	− 0.211	− 0.519	0.096	
Postgraduate/doctorate degree		− 0.101	0.080	− 1.253	0.210	− 0.236	− 0.605	0.133	
TFEQ_Uncontrolled_eating		0.007	0.003	2.632	0.009	0.088	0.022	0.153	***
TFEQ_Cognitive_restriction		0.015	0.003	4.382	< 0.001	0.136	0.075	0.197	***
TFEQ_Emotional_eating		0.002	0.004	0.535	0.593	0.024	− 0.065	0.113	
IES_Unconditional_Permission _to Eat		− 0.256	0.017	− 14.858	< 0.001	− 0.477	− 0.540	− 0.414	***
IES_Eating for Physical Rather than Emotional Reasons		0.028	0.020	1.393	0.164	0.062	− 0.025	0.148	
IES_Reliance on Hunger and Satiety Cues		− 0.007	0.016	− 0.442	0.659	− 0.012	− 0.067	0.042	
IES_Body-Food Choice Congruence		0.131	0.016	8.327	< 0.001	0.249	0.191	0.308	***
MHC_Social_well-being		0.002	0.003	0.931	0.352	0.030	− 0.033	0.092	
MHC_Emotional_well-being		− 0.008	0.004	− 1.777	0.076	− 0.061	− 0.129	0.006	*
MHC_Psychological_well-being		− 0.002	0.002	− 0.672	0.502	− 0.026	− 0.101	0.049	
Constant		1.514	0.179	8.478	< 0.001				***
Mean	1.53				SD dependent var		0.427		
dependent var									
R-squared	0.491				Number of obs		922		
F-test	51.259				Prob > F		0		
Akaike crit	6322.16				Bayesian crit. (BIC)		6409.038		
(AIC)									

*** p < 0.01, ** p < 0.05, * p < *0.1*

*ONI: orthorexia_nervosa_inventory; TFEQ: three‑factor_eating_questionnaire, IES: intuitive_eating_scale‑2, MHC:mental_health_continuum*

The model revealed that when controlling other variables, Unconditional Permission to Eat, Body-Food Choice Congruence, Cognitive Restriction, Uncontrolled eating and age were significant predictors of ONI composite factor. The regression indicated that except for IES-UPE, effect sizes were small or negligible (Table 4*)*. Overall, our analysis confirmed that ONI-Hu measures concepts that are distinct from or not outliers compared to existing measures, justifying its methodological use.

## Discussion

### Factorial structure and reliability of ONI-Hu

We aimed to assess the psychometric properties of the Hungarian version of the Orthorexia Nervosa Inventory. The CFA provided support for the original three-factor structure. Both ONI-Hu and the other questionnaires demonstrated acceptable reliability in our sample. The associations with other key variables (such as TFEQ, IES, and MHC) support ONI’s convergent and discriminant validity. The composite ONI score was positively associated with the TFEQ Cognitive Restriction and negatively with the overall Intuitive Eating Scale, but indicated no significant relationship with mental health indicators. The original three-factor structure, which has been consistently validated in previous studies, including a recent one using a clinical sample [44, 45, 56], was confirmed, showing strong internal consistency.

The three factors represent areas where orthorexia-related or orthorexia-caused problems emerge. Examples include rigid behavior and rituals related to food and eating (Behaviors), disturbances of emotional life (Emotions), impairments of social life, and physical issues that arise due to the strict dietary rules (Physical and psychosocial impairments). The three factors align with the notion that to capture the complexity of the problem and distinguish between healthy eating and ON, it may be necessary to examine all three dimensions [5, 43].

The ONI composite factor and the Behaviors scale strongly correlate with each other. This suggests that behavioral characteristics are most prominently expressed in ON or that the individuals with ON are more aware of their behavior-related symptoms. Furthermore, they often take pride in their healthy eating habits.

Conversely, the distress and anxiety they experience might not feel as intense, they might not be as aware of them, or they might not associate them as much with their relationship with eating [57]. ON is an ego-syntonic disorder, and prior research on ED has shown that increased severity of the disorder is often associated with a lower level of insight about the disorder [58] and that the preoccupations and rituals are usually perceived as ego-syntonic by individuals with ED diagnosis, recovered patients and restrictive-eating individuals [59, 60].

The associations with key variables support ONI-Hu’s convergent and discriminant validity. ONI scores positively correlated with TFEQ Cognitive Restriction and negatively correlated with the IES Unconditional Permission to eat—consistent with previous findings indicating a positive association between ON tendency and disordered, especially restrictive eating [43, 44]. Furthermore, no significant correlation was found between ONI composite score and the TFEQ Emotional eating and Uncontrolled eating. These findings support the convergent validity and suggest that ONI-Hu effectively captures the restrictive nature of orthorexia nervosa.

According to past research, there are numerous similarities between ON and anorexia nervosa [3, 61], and in fact, AN often transitions into ON, especially during recovery attempts [57]. The restrictive, overcontrolling eating behavior observed in individuals with ON, may be connected to the perfectionism that is often characteristic of those with orthorexia as well [4, 61]. Moreover, the perfectionism and associated unrealistic standards that individuals with ON tendencies set for themselves—such as overly strict rules regarding eating in the case of ON—often escalate over time [62].

The ritualized behaviors in ON bear similarities to OCD [61], while the excessive preoccupation with rules, inflexibility, and perfectionism share resemblances with OCPD [4]. Rituals and preoccupation might contribute to establishing a sense of control and security. Therefore, the orthorexic eating behaviors—similar to other EDs—may function as a coping mechanism [62].

The association of ONI with other covariates, such as the MHC scale or the other TFEQ subscales (Emotional eating and Uncontrolled eating), supports the discriminant validity since the results only indicated small effect sizes or nonsignificant relationships. The findings show that there is no significant relationship between the ONI composite factor and aspects of mental health. This may result from the positively and negatively correlated MHC and ONI subscales, thus being mutually suppressive for the overall scale.

A possible explanation for the positive association between orthorexic behaviors and mental health might be that the behaviors and preoccupations associated with ON provide a sense of control to the individual [63]. The sense of control could lead to a distorted perception of well-being. Another suggestion could be that individuals with ON believe that they adhere to a healthy diet and that their eating habits enable them to achieve perfect physical health [1]. Additionally, they often fail to recognize the extent of harm caused by the disorder. For example, they may not realize how much weight they have lost, how little energy they possess, or how distant they have become from their peers or family members [57]. It is also possible that the impairment of social life does not necessarily result in complete isolation but rather a shift towards interacting with individuals who share similar issues [64].

Furthermore, individuals with ON frequently experience positive emotions related to their condition. Recovered orthorexic individuals reported excitement when imposing new dietary rules and fantasizing about possible outcomes [65]. Moreover, receiving flattery and recognition from their environment (which may come from their peers with similar problems) could contribute to the perception of well-being despite experiencing anxiety or worry related to their eating habits [57, 65].

It was also observed that individuals with ON, in contrast to their previous issues, such as addictions or other eating disorders, perceive their preoccupation with healthy eating as caring about themselves [57], which also contributes to the distortion of their well-being. A form of self-deceptive behavior is frequently associated with ON, particularly during recovery from other EDs, where „healthy” eating habits may appear less problematic both to the individual and their surroundings, allowing the disorder to manifest differently and, therefore, to persist [57].

Furthermore, these findings not only deepen our understanding of ON but also further strengthen the validity of ONI-Hu. Based on the results, we suggest that the ONI-Hu factors capture distinct aspects of the disorder despite their strong correlation. The regression model results indicate that the convergent and discriminant factors explain almost 50% of the variance in ONI scores when controlling for demographic variables. However, individually, none of them significantly contributed to the explained variance, which further supports the validity of ONI-Hu.

### ON associations with other variables

We investigated the relationship between ON, disordered eating, and intuitive eating. The correlation between emotional distress related to ON and disordered eating behaviors suggests that the rigid dietary rules may serve to manage low self-esteem by keeping away self-loathing thoughts, feelings of self-hatred, and shame. Case studies have documented instances where individuals with ON tendencies experienced self-loathing and panic attacks when they were unable to adhere to their strict dietary rules [57]. Shame, both as a contributing factor and as a consequence, has been well-documented in patients with eating disorders [66, 67].

These findings support the idea that restrictive eating behavior in ON functions as a coping mechanism, similar to other eating disorders, such as anorexia nervosa [66]. Restrictive eating often provides a false sense of control, empowerment, pride, or invincibility, and protection from negative emotions like shame or uncertainty. Case studies highlighted that instability and feelings of loss of control, often caused by significant life changes, such as moving away to college or getting a chronic illness diagnosis, usually precede the development of orthorexic habits [63].

Cognitive restriction of eating and Uncontrolled eating were positive predictors of ON tendencies, while Unconditional permission to eat scale of IES-2 was a negative predictor. These results suggest that loss of control and deviation from dietary rules also manifest in ON, similar to other EDs. This might lead to increased restriction and subsequent episodes of losing control over eating—which has also been described in other EDs and the past years in orthorexic individuals as well [65, 66, 68].

Furthermore, studies on executive functions and ON might support this idea. Results indicate that higher orthorexic symptoms correlate with lower levels of various executive functions, such as self-monitoring, working memory, set-shifting, emotional inhibition and control, task planning, initiation, and monitoring [61, 69]. In addition, all-or-nothing thinking or catastrophic thinking, often present in ED-s, may also contribute to the loss of control due to the additional psychological distress they create [57, 65, 70].

ONI-Hu factors showed weak to moderate negative associations with most of the IES subscales, including the overall IES. However, we observed a positive, moderate relationship between ONI Behaviors, the ONI composite factor, and the IES Body-Food-Choice Congruence subscale. This may suggest that individuals with ON tendencies may be more inclined to adhere to a standard or belief regarding what is healthy rather than attuning to their body’s signals. The negative associations between other IES scales and ON tendencies support this idea. Additionally, these findings may be linked to traits such as perfectionism, OCD, or OCPD traits, which are commonly present in ON [4, 61].

## Limitations

The limitations of our study include the fact that the questionnaires were predominantly distributed among individuals interested in healthy eating. Furthermore, there was an imbalance in gender and other demographic indicators. Convenience sampling and the non-representative sample limit our ability to generalize the results to a broader population. Retesting the ONI-HU on a more diverse sample in future research may be worthwhile to obtain more accurate results.

The cross-sectional design of our study prevents us from inferring causality, and we did not examine the temporal stability of the factors. Additionally, participants were required to complete long questionnaires, potentially contributing to dropout bias [71]. Additionally, we did not conduct prior screening for past or current eating disorders. Upon accepting the informed consent form, participants were required to declare that they had not been treated for any neurological or psychiatric disorders at that time or in the past. Of course, this does not exclude individuals who were unaware of being affected by an eating disorder. In future research, it may be beneficial to include questions about past or current eating disorders in the questionnaire package.

The ONI Composite factor and ONI behaviors strongly correlated with each other, which could raise several concerns. However, definitions related to ON highlight all three dimensions included in the ONI-Hu. Furthermore, the reliability of the other two scales is adequate, with items loading well onto their factors. Since few studies used the ONI and its translated versions, and we do not yet have diagnostic criteria, we do not have a clear view. In the future, item-response analysis could bring us closer to a deeper understanding of the nature of orthorexia, as well as which measurement tools could most reliably assess the disorder.

Furthermore, there are also some issues regarding another questionnaire, the IES-2. The questionnaire includes positively and negatively worded items, potentially confusing the participants [72]. Current recommendations suggest that questionnaires should be developed with positively worded items, which align more effectively with the measured theoretical construct [73]. In future research, it may be beneficial to use the revised IES-3, which has been developed based on these recommendations [74].

In the future, it would be helpful to examine the role of control in individuals with high orthorexic tendencies. Additionally, future research could explore the associations between emotion regulation difficulties, impairments with self-regulation, and orthorexia. An essential question to address is why specific individuals with orthorexic tendencies experience episodes of loss of control. Future directions should include how individuals with orthorexia relate to feelings of uncertainty and shame and assess the potential role these emotions play in the development and maintenance of the disorder. Furthermore, exploring the relationship between orthorexia and various dimensions of mental health would also be necessary, particularly in light of conflicting findings in past research. Beyond these aspects, testing the validity of the ONI-Hu in clinical samples could also be one of the following steps.

## Conclusion

The ONI-Hu questionnaire measures characteristics of orthorexia nervosa and demonstrates good reliability indicators in its original version and the Hungarian adaptation. In our study, the original three-factor structure was confirmed.

Consistent with prior research, the ONI-Hu effectively captures the restrictive nature of orthorexia nervosa. Despite the variability in mental health indicators, orthorexic behavior is associated with significant emotional distress and impairments in physical and psychosocial aspects of life. Additionally, it offers a more detailed understanding of the disorder’s nature compared to previously developed measurement tools available in Hungarian. It assesses behavioral, emotional, and psychosocial problems related to orthorexia and measures the presence of physical impairments often associated with the disorder.

## What is already known on this subject?

Regarding the existing Hungarian questionnaire for measuring ON, reliability issues arose, and it also does not cover the wide range of problems associated with orthorexia to the same extent as ONI-Hu does.

## What does this study add?

The ONI-Hu is a reliable questionnaire that captures orthorexia nervosa, and is aligned with the recently developed proposed diagnostic criteria. Additionally, the results might help in gaining a deeper understanding of the nature of this disorder.

## Data Availability

The Ethics Approval for the study did not include the permission to make the dataset generated and analysed publicly available. However, the datasets generated and/or analysed during the current study are available from the corresponding author on request.
